# Employees and the National Insurance Act

**Published:** 1912-05-11

**Authors:** 


### NATIONAL INSURANCE ACT DEPARTMENT.

### EMPLOYEES AND THE NATIONAL INSURANCE ACT.

### X.—Arrears of Contribution.

It is by no means an uncommon occurrence for a life assurance company to receive a letter from one of its old policyholders in which he states that he has paid the premiums for, say, thirty years, *without ever missing a payment.* Some policyholders make such a statement with pride, others do so thinking to strengthen the grounds of their application for some special benefit or consideration from the company. Both classes alike fail to appreciate the fact that they would not now be paying premiums unless payment had been made each time a premium became due. If the premium is not paid the assured is in "arrear," and he must make it good before any further premium can be accepted by the company. There is, therefore, no special virtue in the plea of *never having missed a payment.* On the other hand, there are manifest disadvantages of failing to make the payments when due. Omission to pay means the lapsing of the policy, and consequent suspension from benefit, though when a policy has been some years in force and a value attaches to it, it may, either automatically by virtue of its conditions, or at the option of the assured, be converted into a free policy for a reduced benefit. These conditions attaching to a life assurance policy are, doubtless, familiar to most of our readers, and it is for that reason we have chosen life assurance as an example to emphasise the point that in order to obtain the full benefit under an insurance contract the insured must pay the stipulated contribution *not sometimes only, but each time it becomes due*, and to illustrate how arrears of contribution arise.

#### Lapsed Insurances.

Sickness insurance, as administered by the Friendly Societies, is very like life assurance in this respect. Rather more latitude would seem to be allowed in the matter of punctuality of payment, and in many societies it is known that a period of something like six months is allowed to elapse, since the first contribution became due which was not paid, before the member is struck off the books as being out of benefit. The arrears of contribution must be paid, together with such fines as may be imposed by the rules of the society, before the contract of insurance can be reinstated on its original basis. Both in life assurance and sickness insurance, as at present in vogue, the contract does not bind the insured to pay the contributions. Payment is quite optional on his part. He may in fact discontinue payment merely from caprice or, as is more often the case, through inability to pay due to unemployment or other adverse circumstances. This question of arrears of contribution is a very difficult one. On the one hand, the insurer—whether a life office or a Friendly Society—must receive the necessary consideration for the risk undertaken, while, on the other hand, the insured may be overtaken by adverse circumstances which render the continuation of payments impossible, and he feels the loss of his former payments from which at such times he is inclined to think he has received no benefit. This is one of the inevitable consequences of the voluntary nature of the contract, and it is impossible to give full satisfaction to all parties concerned.

#### How Arrears Accrue.

It may, perhaps, be thought that under a compulsory system of insurance, such as that provided by the National Insurance Act, no such difficulty will arise, but, unfortunately, this is not so. The employed contributor is compulsorily insured whilst employed, and his contributions and those of the employer are compulsory payments, so that mere caprice does not enter into the question, and the approved societies administering the Act will in consequence be relieved of the necessity of writing on and off their books numberless cases of discontinuance arising from no apparent cause. Although this cause of annoyance will not exist there will still be a large number of cases in which the premiums or contributions will not be paid. The occasion will arise when the insured person is temporarily out of employment, and having no employer for the time being he will either himself have to pay the joint contribution of the employer and employee or fall into arrear with his payments. The joint contribution in the case of a man is 7d. a week and for a woman 6d. per week, and these charges will press heavily on such funds as are available for maintenance of the person, who normally does not receive more than £160 per annum and possibly much less, when there is no current income from the usual employment. In such circumstances arrears are bound to accrue, and to meet the case elaborate provision has been made in the Act with a view to safeguarding the interests of all parties. It is clear from what has already been said that with contributions in arrear the approved societies will not be able to pay full benefits, while at the same time, by reason of the fact that sickness increases with age, the society will hold a reserve accumulated from past payments, which must in equity be used, so far as it will go, in providing reduced or modified benefits.

There is a further complication to be met. When employment is resumed contributions will again become payable compulsorily, and thus under the National Insurance Scheme *it will be possible for a person to be insured who has not paid always without missing.* Here, again, the benefit must be modified in order to preserve equality in value as between the benefit on the one hand and the contributions on the other. The insurance of persons who have not paid all the contributions is a new feature in insurance practice, and the method of dealing with the situation is both novel and interesting. The general provisions governing the reduction of benefits when contributions are in arrear are set out in seven subsections under section 10 of the Act, and the Fifth Schedule furnishes the modified rates of sickness benefit as they apply according to the average amount of the arrears.

### Reduction or Modification of Benefits.

The modification of the benefits does not directly depend upon the total amount of the arrears, but upon the average yearly amount. Thus, supposing four years have elapsed since the insured person first became a member of an approved society and that during such period he had been unemployed for fifty-two weeks and did not himself pay the contribution, the contributions will in such circumstances be a year in arrear, but as the person has been a member of the society for four years the average amount of his arrears will be thirteen weeks.

If membership had continued for thirteen years and one year's contributions had not been paid, the average amount of arrears would be four weeks. This is only a rough illustration of the fundamental principle underlying the method of procedure. In point of fact there will be very few cases where the average will work out at an exact number of weeks, and to govern this and other points of detail the precise method of calculation will be prescribed by the Insurance Commissioners.

### Calculation of Arrears—Exemptions.

In calculating the arrears of contributions no account is to be taken of any accruing in respect of any period during which the insured person has been in receipt of sickness or disablement benefits. Thus when the insured person is rendered incapable of work by some specific disease, or by bodily or mental disablement, and is in consequence unable to follow his employment, no contributions will become payable, and no account is to be taken of the payments omitted during such incapacity in calculating the average amount of arrears. A similar exemption from payments is provided in the case of a woman who is herself an insured person, and entitled to maternity benefit during two weeks before and four weeks after the birth of her child. Again, in the case of maternity benefit payable in respect of the posthumous child of an insured person no account is to be taken of the contributions unpaid during the period subsequent to the father's death. In the case of an employed contributor no account is to be taken of arrears accruing during the first twelve months after the Act comes into operation. It is to be noted that this last exemption does not extend to voluntary contributors. Apart from these specific exemptions, the contributions are deemed to be payable in respect of every week from the date of entry into insurance.

### Rates of Reduction of Benefit.

When an insured person is in arrear to an amount greater than thirteen weekly contributions a year on the average, his right to sickness and disablement benefits will be suspended, and when the arrears are greater than twenty-six weekly contributions on the average, the right to medical, sanatorium, and maternity benefits will also cease. When the arrears are less than four weekly contributions a year on the average, the full rate of benefit applicable to the case becomes payable. If, however, the contributions are four weeks or more in arrear on the average for each year since entry into insurance, the rate of sickness benefit will be reduced by 6d. a week for men and 3d. a week for women, for every complete week the contributions are in arrear above three weeks. This reduction continues until the rate of sickness benefit is reduced to 5s. a week, and after that the commencement of benefit is to be postponed by one day for every such week of arrears. An example will make the position clearer. Let it be supposed that the employed contributor, apart from the arrears, would have been entitled to the full rate of benefit. If the contributions are seven weeks in arrear on the average, the rate of sickness benefit for a man will be 8s., that is the full standard rate of 10s. less four times 6d. In the case of a woman the benefit will be 6s. 6d., that is 7s. 6d. less four times 3d.

The rates of reduction applicable to voluntary contributors are not stated in the Act, but they will be prescribed by the Insurance Commissioners in due course.

The option to pay up the arrears which have accrued during the calendar year current at the date of payment and the previous year is allowed, but arrears which accrued due at any earlier date can apparently never be paid off. If at the time of paying up the arrears the insured person is incapable of work, or if he becomes ill within a month of such payment, he will be considered to be in arrear until the month has elapsed.

The arrears of contribution, so far as they relate to the amount that would have been paid by the employer if the member had continued in his last employment, may be excused by any approved society if it thinks fit.

These provisions as to arrears have probably given the reader the impression of being very complicated, and so, in fact, they are, taken as a whole, but it will be fairly easy to fit them to any particular case. The general gist of the matter is that arrears mean reduction of benefit, and consequently an insured person who falls out of employment would do well to consider whether some small present sacrifice in keeping up the contributions will not be more than repaid in subsequent years in the form of increased benefit.

### Insurance Questions and Answers.

We have received a letter from Nurse R., Chorley, which raises so many important points of general interest that we propose to devote our article next week to answering her inquiries. The letter is just the sort we want, and we should like to have more similar to it.

NOTE.—Questions and Correspondence on all doubtful points are invited, and should be addressed to The Insurance Editor, The Hospital, 28 Southampton Street, Strand, W.C.

